# Influence of incisor display and lower lip height on smile esthetics and perceived age

**DOI:** 10.1590/2177-6709.31.2.e2625302.oar

**Published:** 2026-06-22

**Authors:** Juliana do Espírito Santo RUSSO, Rafael Cunha BITTENCOURT, Daniella Mascarenhas Calixto BARROS, Rebeca Passeri de ALMEIDA, Diego Coelho LORENZONI, Sergio Luiz MOTA-JÚNIOR, Claudia Trindade MATTOS

**Affiliations:** 1 Universidade Federal Fluminense, Dental School, Department of Clinical Dentistry (Niterói/RJ, Brazil).; 2 Universidade Federal do Rio de Janeiro, Dental School, Department of Pediatric Dentistry and Orthodontics (Rio de Janeiro/RJ, Brazil).

**Keywords:** Dental esthetics, Incisor, Aging, Orthodontics, Estética dentária, Incisivo, Envelhecimento, Ortodontia

## Abstract

**Objective:**

This study investigated the perception of smile attractiveness and age estimation based on different proportions of maxillary and mandibular incisor exposure, combined with changes in lower lip vermilion height.

**Methods:**

Smile photographs were digitally modified to create images with varying proportions of incisor exposure (100%, 80%, 60%, 40%, and 20% maxillary incisor exposure) and reduced or unaltered lower lip vermilion height. A total of 162 evaluators, including orthodontists, dentists, and laypersons, rated attractiveness using a 10-point visual analog scale and estimated the apparent age range (<30, 30-50, >50).

**Results:**

Maxillary incisor exposure significantly affected smile attractiveness, with higher exposure levels (100% and 80%) receiving higher attractiveness scores across all evaluator groups (p < 0.05). Reduction in lower lip vermilion height did not result in statistically significant differences in attractiveness ratings or age estimation (p > 0.05). Orthodontists assigned significantly lower attractiveness scores than dentists and laypersons (p < 0.05). Increased mandibular incisor exposure was significantly associated with the perception of an older apparent age (p < 0.05). Older laypersons demonstrated greater tolerance toward smiles with increased mandibular incisor exposure (p < 0.05).

**Conclusions:**

These findings suggest that the proportion of maxillary and mandibular incisor exposure plays a significant role in the perception of smile attractiveness and age estimation. Furthermore, they highlight the importance of considering individual preferences in dental treatment planning. The results of this study have direct implications for clinical practice in Orthodontics and Esthetic Dentistry, providing valuable guidance for treatment planning and patient communication.

## RESUMO

**Objetivo:**

Este estudo investigou a percepção da atratividade do sorriso e a estimativa de idade com base em diferentes proporções de exposição dos incisivos superiores e inferiores, combinadas com alterações na altura do vermelhão do lábio inferior.

**Material e Métodos:**

Fotografias de sorrisos foram modificadas digitalmente para criar imagens com diferentes proporções de exposição dos incisivos (100%, 80%, 60%, 40% e 20% de exposição dos incisivos superiores) e com altura do vermelhão do lábio inferior reduzida ou inalterada. Um total de 162 avaliadores, incluindo ortodontistas, cirurgiões-dentistas e leigos, classificou a atratividade utilizando uma escala visual analógica de 10 pontos e estimou a faixa etária aparente (<30, 30-50, >50).

**Resultados:**

A exposição dos incisivos superiores afetou significativamente a atratividade do sorriso, sendo que níveis mais elevados de exposição (100% e 80%) receberam maiores escores de atratividade em todos os grupos de avaliadores (p < 0,05). A redução da altura do vermelhão do lábio inferior não resultou em diferenças estatisticamente significativas nas classificações de atratividade ou na estimativa de idade (p > 0,05). Os ortodontistas atribuíram escores de atratividade significativamente menores do que os cirurgiões-dentistas e os leigos (p < 0,05). O aumento da exposição dos incisivos inferiores associou-se significativamente à percepção de uma idade aparente mais avançada (p < 0,05). Leigos mais velhos demonstraram maior tolerância em relação a sorrisos com maior exposição dos incisivos inferiores (p < 0,05).

**Conclusões:**

Esses achados sugerem que a proporção de exposição dos incisivos superiores e inferiores desempenha papel significativo na percepção da atratividade do sorriso e na estimativa da idade. Além disso, destacam a importância de considerar preferências individuais no planejamento do tratamento odontológico. Os resultados desse estudo têm implicações diretas para a prática clínica em Ortodontia e Odontologia Estética, fornecendo orientação valiosa para o planejamento terapêutico e a comunicação com o paciente.

## INTRODUCTION

Smile esthetics has gained significant attention in recent decades due to professionals recognizing the importance of understanding laypersons perception, as these are potential dental patients.^1^ ^,^ ^2^ Their views on smile characteristics affecting esthetics should be valued, as they may influence treatment needs ranking and orthodontic planning.^2^ ^,^ ^3^ Recent survey-based studies highlight that laypeople perceive an attractive smile as relevant to social life and communication.^4^

Orthodontists typically have more critical esthetic evaluation of smile and occlusal characteristics, compared to laypersons and other dental professionals,^5^ ^,^ ^6^ making recognition of patients’ perception fundamental. Evidence suggests that laypersons prioritize global smile harmony and easily perceived features (e.g., alignment, symmetry, and tooth/gingival display), while clinicians tend to be more critical of subtle deviations.^1^ ^,^ ^2^ ^,^ ^5^ ^,^ ^7^ ^-^ ^10^

Previous research indicates that incisor exposure affects smile esthetics.^6^ ^,^ ^11^ Ideal maxillary incisors exposure in young individuals is approximately 3-5 mm at rest and 10 mm during posed smiles.^12^ ^,^ ^13^ With aging, the upper lip becomes more flaccid, and lip vermilion height, particularly in the lower lip, decreases.^14^ ^-^ ^17^ Additionally, maxillary incisor exposure decreases while mandibular incisor exposure increases proportionally in both males and females,^12^ ^,^ ^16^ ^,^ ^18^ ^-^ ^20^ causing smiles to become shorter and wider.^21^

Orthodontic treatment can influence final incisor position, requiring special care when altering vertical position through extrusion or intrusion. Such changes can impact incisor exposure and overall smile esthetics.^13^ ^,^ ^19^ In young adults, maxillary incisor exposure at rest is commonly reported to range from approximately 2 to 4 mm, decreasing with age. During a posed smile, full display of the maxillary incisor crowns, with minimal or no gingival exposure, is generally considered esthetically favorable and serves as a reference for smile design and orthodontic treatment planning.^12^ ^,^ ^13^ ^,^ ^19^ ^,^ ^20^

Aging has also been reported as influencing smile esthetics perception, with younger age groups preferring greater incisor exposure and older individuals preferring less incisor display.^22^ Moreover, perceived age is a relevant outcome, as smile characteristics may influence social perception, self-esteem, and the subjective experience of appearing older or younger. Recently, esthetic research has expanded beyond maxillary-focused and static smile analyses to include greater attention to mandibular anterior incisor display and dynamic smile assessment.^23^ However, there is notable lack of studies evaluating esthetic perception of different maxillary and mandibular incisor exposure proportions in smiles, considering whether these differences influence perceived age when assessed by professionals and laypersons.

Our primary objective was to investigate smile attractiveness perception among orthodontists, dentists, and laypersons concerning varying incisors exposure proportions coupled with reduced or unaltered lower lip vermilion height. The secondary objective was to assess if the proposed changes impact perceived age range of the smile’s owner, and to assess whether age, sex and group influenced the scores attributed to each image.

The null hypothesis was that variation in incisor exposure proportions and lower lip vermilion height do not influence smile attractiveness perception or perceived age estimation among orthodontists, dentists, and laypersons, and that evaluators’ age, sex, and professional group would not significantly affect the scores assigned to each image.

## MATERIAL AND METHODS

This research project was approved by the Research Ethics Committee of Faculdade de Medicina da Universidade Federal Fluminense (CAAE # 70685923.1.0000.5243). Written informed consent was obtained from photographic models and research participants.

This study utilized an extraoral frontal smile photograph with half-open mouth of a 24-year-old female volunteer, with no considerable facial asymmetry, and an intraoral frontal photograph of a 22-year-old female volunteer with well-aligned teeth. Both models provided written informed consent.

Photographs were modified using GIMP 2.10.32 free open-source software (The GIMP Development Team, Berkeley, California, USA). The extraoral photograph was standardized by removing distracting factors such as skin signs, irregularities and spots, with the edited side mirrored to ensure smile symmetry. The dentition within the smile area was cropped, and the lower lip was cropped by a 1mm-strip to create an additional smile with shorter-vermilion lower lip. The intraoral frontal photograph was edited by mirroring the right side for symmetry, then layered behind the extraoral cropped image and proportioned according to clinical measurements from the model, allowing subsequent manipulation.

**Figure 1:**
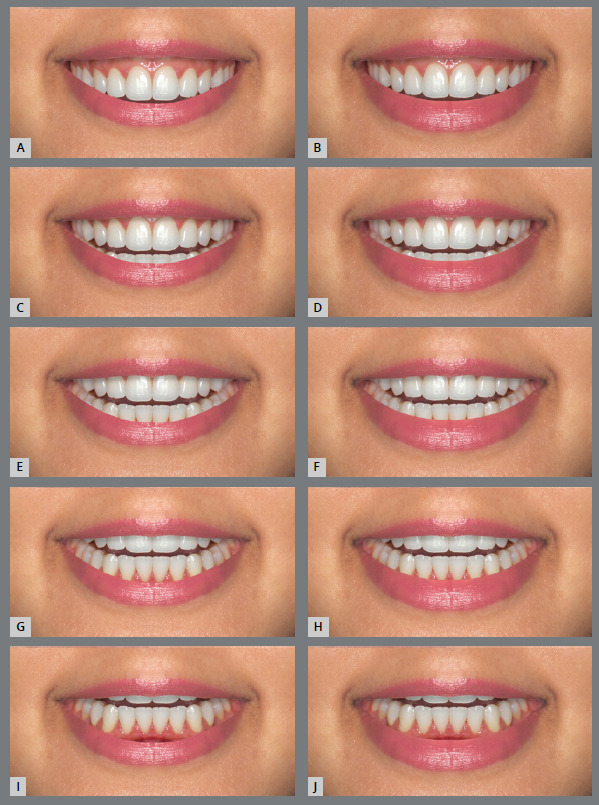
Smiles with manipulated maxillary and mandibular incisor exposure; reduced lower lip vermilion height (left column) and unaltered lower lips (right column). **A** and **B**, 100% upper incisor exposure with no lower incisor display (100Ui); **C** and **D**, 80% upper/20% lower incisor exposure (80Ui-20Li); **E** and **F**, 60% upper/40% lower incisor exposure (60Ui-40Li); **G** and **H**, 40% upper/60% lower incisor exposure (40Ui-60Li); **I**and **J**, 20% upper/ 80% lower incisor exposure (20Ui-80Li).

Ten images were created with alterations in vertical position of the intraoral photograph, displaying varying proportions of upper incisors (Ui) and lower incisors (Li) exposure: 100% Ui exposure (100Ui), 80% Ui and 20% Li exposure (80Ui-20Li), 60% Ui and 40% Li exposure (60Ui-40Li), 40% Ui and 60% Li exposure (40Ui-60Li), and 20% Ui and 80% Li exposure (20Ui-80Li), for both unaltered and reduced lower lip scenarios (Fig. 1). Incisor exposure was calculated in pixels by measuring vertical display of right central maxillary and mandibular incisors and calculating each percentage to the vertical distance between lower border of upper lip and upper border of lower lip, measured over a line passing through the middle of maxillary right incisor crown.

Using Qualtrics XM online survey platform (Qualtrics, Provo, Utah, USA), participants first previewed the 10 images assembled, then evaluated them individually in random order. Smile attractiveness was assessed using a digital 10-point visual analog scale (VAS), with endpoints representing “most unattractive” at zero on the left and “most attractive” at 10 on the right. For descriptive purposes, a VAS score of 5 was adopted as the threshold to categorize smiles as unattractive (<5) or attractive (≥5).^24^ Participants could slide the cursor to any point along the scale. Participants also estimated the smile’s owner age range for each image marking one of three multiple-choice answers: less than 30 (<30), 30 to 50 (30-50), or above 50 (>50). Prior to data collection, the questionnaire and image set were reviewed by three senior orthodontists to ensure clarity, relevance, and content validity of the survey instrument. The online questionnaire link was sent to evaluators fulfilling inclusion criteria, who responded between January and April 2024. 

Evaluators were divided into three groups with inclusion criteria: 1) orthodontists should be either orthodontic residents or specialists; 2) dentists should have university degree without orthodontic training; and 3) laypersons should have completed high school and be above 18 years. Demographic data collected from participants included age and sex, and additional data regarding educational level or professional experience were not collected. All 162 volunteers signed online informed consent. Participants were recruited through convenience sampling using professional networks and direct invitations, and eligibility was verified prior to participation based on self-reported information.

Sample size was calculated considering α of 5% and β of 20%, standard deviation from a previous study,^25^ and minimum detectable difference of 1 score, resulting in 54 participants per group, totaling 162 individuals.

Eighteen participants were invited to retake the questionnaire after one month to estimate method reliability. The recall sample comprised 6 orthodontists, 6 dentists, and 6 laypersons, who were selected by convenience from the original sample to ensure equal representation of the evaluator groups.

## STATISTICAL ANALYSIS

Data were analyzed using Jamovi Project (2024, version 2.5), retrieved from https://www.jamovi.org. Age differences and sex distribution among evaluator groups were tested using Kruskal-Wallis and chi-square tests, respectively. Scores for each image were represented through boxplots with calculated means and standard deviations. Repeated-measures ANOVA, followed by Tukey’s *post-hoc* test assessed score differences for each image, considering evaluator group as factor. Age ranges frequencies attributed for each image were presented in bar plots, with intergroup differences assessed using chi-square tests. Friedman test with Durbin-Conover pairwise multiple comparison method was used for intragroup comparison of different images and Kruskal-Wallis test with the Dwass-Steel-Critchlow-Fligner pairwise multiple comparison method for intergroup comparison examining median differences, considering age range as categorical variable. Linear regression analysis tested the influence of evaluators’ age as continuous variable, and sex and group as categorical variables (independent variables) on scores attributed to each image (dependent variable), with separate models run for each image. One-way intraclass correlation coefficient (ICC) for absolute agreement was used to assess method reliability for all images. P-values below 0.05 were considered statistically significant.

## RESULTS

A total of 162 participants completed the questionnaire: 54 orthodontists (43 females, 11 males, mean age 34.22 ± 9.12 years), 54 dentists (37 females, 17 males, mean age 36.26 ± 9.69 years), and 54 laypersons (35 females, 19 males, mean age 36.15 ± 10.23 years). All orthodontists who participated in the study had completed formal dental training. No significant differences were observed in age (p = 0.103) or sex distribution (p=0.210) between groups.

The ICC value was 0.826, confirming measure reliability.

Table 1 presents mean attractiveness scores assigned to each image. The highest scores were given to images with 100 and 80% upper incisor exposure. Mean scores decreased progressively for images with 60%, 40%, and 20% upper incisor exposure, with significant differences among them.

**Table 1:** Mean (CI ) values attributed to each image by groups of examiners, with intragroup statistical differences

	Orthodontists			Dentists			Laypersons		
	Reduced LLH	Unaltered LL	p-value	Reduced LLH	Unaltered LL	p-value	Reduced LLH	Unaltered LL	p-value
100 Ui	6.23 (5.65-6.81)^A^	7.43 (6.93-7.93)^A^	<0.001	6.56 (5.90-7.22)^AB^	7.68 (7.18-8.18)^A^	<0.001	6.48 (5.76-7.20)^AB^	7.53 (6.93-8.13)^A^	<0.001
80 Ui - 20 Li	6.63 (5.97-7.29)^A^	7.06 (6.42-7.69)^A^	0.086	7.45 (6.92-7.98)^A^	7.41 (6.87-7.95)^A^	0.874	7.52 (6.97-8.07)^A^	7.78 (7.27-8.28)^A^	0.269
60 Ui - 40 Li	4.45 (3.89-5.01)^B^	4.15 (3.65-4.65)^B^	0.109	5.39 (4.81-5.97)^B^	5.24 (4.64-5.85)^B^	0.593	5.77 (5.07-6.47)^B^	5.55 (4.87-6.23)^B^	0.379
40 Ui - 60 Li	2.14 (1.66-2.62)^C^	2.34 (1.89-2.79)^C^	0.323	3.52 (2.97-4.08)^C^	3.39 (2.89-3.89)^C^	0.455	3.54 (2.91-4.17)^C^	3.60 (2.99-4.21)^C^	0.790
20 Ui - 80 Li	1.24 (0.86-1.63)^D^	1.24 (0.92-1.56)^D^	0.964	2.05 (1.55-2.54)^D^	2.17 (1.64-2.69)^D^	0.491	2.42 (1.81-3.03)^D^	2.43 (1.81-3.04)^D^	0.955

CI - 95% confidence interval; LLH - lower lip height; LL - lower lip; A, B, C, D - different letters mean statistical significant difference in the column.

Comparisons between images with reduced versus unaltered lower lips with identical incisor exposure revealed significant difference only for 100% upper incisor exposure across all examiner groups (mean difference of 1.2 for orthodontists, 1.12 for dentists and 1.03 scores for laypersons), with higher scores for unaltered lips.

**Figure 2:**
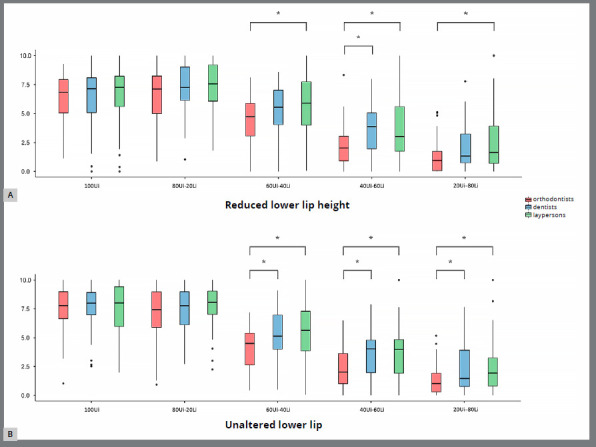
Boxplots showing statistical differences (*) among examiner groups (orthodontists, dentists, laypersons) for images of: A) reduced lips or B) unaltered lips.

Significant differences were observed between orthodontists and laypersons for reduced lips images with 60%, 40% and 20% upper incisor exposure. Similarly, orthodontists differed markedly from other examiner groups in unaltered lips images with 60%, 40% and 20% upper incisor exposure. Orthodontists consistently assigned lower scores (Fig. 2).

**Figure 3:**
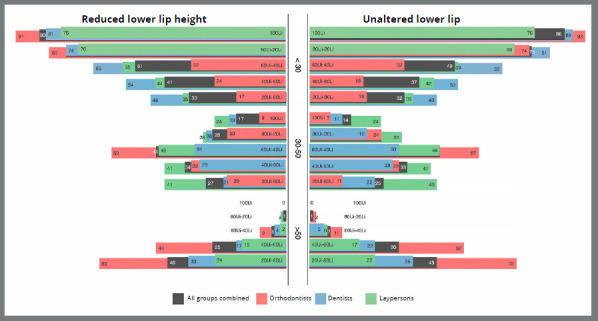
Distribution of perceived age categories (<30, 30-50, >50 years) assigned to each image with reduced or unaltered lower lip height. Horizontal bar plots display the percentage (%) of evaluators who attributed each age category to a given image. Results are presented according to evaluator group (orthodontists, dentists, laypeople) and for all groups combined.

Figure 3 illustrates perceived age frequency distribution assigned to each image by combined examiner groups and each group separately. Images with 100% or 80% upper incisor exposure with both lip conditions were predominantly associated with individuals under 30 by 68.5 to 92.6% of evaluators. Conversely, images with 60%, 40% or 20% upper incisor exposure showed marked differences between orthodontists and other evaluators. Most orthodontists attributed 60Ui-40Li images to individuals aged 30-50 years, and 40Ui-60Li and 20Ui-80Li images to individuals over 50. Most dentists attributed all images to the under-30 category, with percentage attributions for 30-50 and above-50 age categories increasing proportionally to greater lower incisors exposure. Laypersons predominantly associated 60Ui-40Li and 40Ui-60Li images with the under-30 category, while 20Ui-80Li images were more frequently attributed to the 30-50 range. Table 2 presents intragroup and intergroup statistical differences in age attribution.

**Table 2:** Absolute and relative (%) frequency of age ranges attributed to each image by groups of examiners, with intragroup and intergroup statistical difference.

	Orthodontists				Dentists				Laypersons				
	< 30	30-50	>50	Statistical intragroup difference	< 30	30-50	>50	Statistical intragroup difference	< 30	30-50	>50	Statistical intragroup difference	Intergroup difference p-value
Reduced lower lip height													
100 Ui	49 (90.7%)	5 (9.3%)	0 (0%)	A	44 (81.5%)	10 (18.5%)	0 (0%)	A	41 (75.9%)	13 (24.1%)	0 (0%)	A	0.121
80 Ui - 20 Li	43 (79.6%)	11 (20.4%)	0 (0%)	A	40 (74.1%)	14 (25.9%)	0 (0%)	AB	38 (70.4%)	15 (27.8%)	1 (1.9%)	A	0.564
60 Ui - 40 Li	17 (31.5%)	32 (59.3%)	5 (9.3%)	B	35 (64.8%)	17 (31.5%)	2 (3.7%)	AB	30 (55.6%)	23 (42.6%)	1 (1.9%)	AB	0.006*
40 Ui - 60 Li	13 (24.1%)	17 (31.5%)	24 (44.4%)	C	29 (53.7%)	16 (29.6%)	9 (16.7%)	BC	24 (44.4%)	22 (40.7%)	8 (14.8%)	BC	<0.001*
20 Ui - 80 Li	9 (16.7%)	11 (20.4%)	34 (63.0%)	C	25 (46.3%)	11 (20.4%)	18 (33.3%)	C	19 (35.2%)	22 (40.7%)	13 (24.1%)	C	<0.001*
Unaltered lower lip													
100 Ui	50 (92.6%)	4 (7.4%)	0 (0%)	A	48 (88.9%)	6 (11.1%)	0 (0%)	A	41 (75.9%)	13 (24.1%)	0 (0%)	A	0.034*
80 Ui - 20 Li	40 (74.1%)	13 (24.1%)	1 (1.9%)	A	44 (81.5%)	10 (18.5%)	0 (0%)	AB	37 (68.5%)	17 (31.5%)	0 (0%)	AB	0.347
60 Ui - 40 Li	17 (31.5%)	31 (57.4%)	6 (11.1%)	B	35 (64.8%)	16 (29.6%)	3 (5.6%)	BC	27 (50.0%)	24 (44.4%)	3 (5.6%)	BC	0.014*
40 Ui - 60 Li	10 (18.5%)	16 (29.6%)	28 (51.9%)	C	27 (50.0%)	15 (27.8%)	12 (22.2%)	CD	23 (42.6%)	22 (40.7%)	9 (16.7%)	C	<0.001*
20 Ui - 80 Li	10 (18.5%)	6 (11.1%)	38 (70.4%)	C	23 (42.6%)	12 (22.2%)	19 (35.2%)	D	19 (35.2%)	23 (42.6%)	12 (22.2%)	C	<0.001*

A, B, C, D - Different letters indicate statistically significant difference in the column.

**Figure 4:**
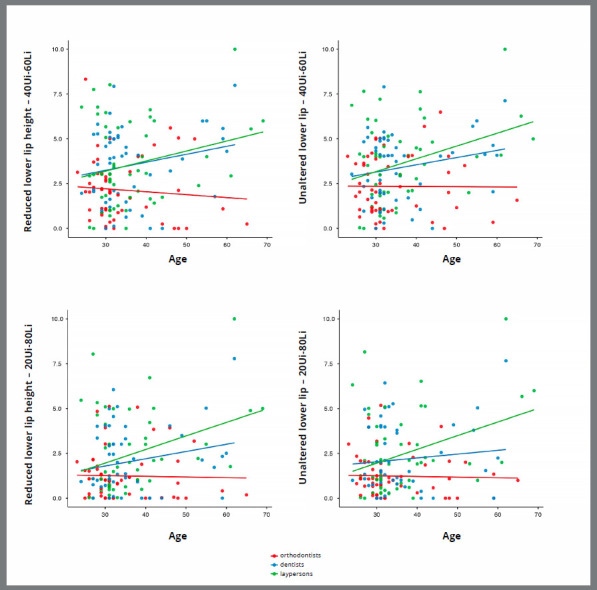
Linear regression scatterplots showing that older evaluators, particularly laypersons, assigned higher scores to images with a greater lower incisor exposure.

Linear regression analysis (Table 3) indicated statistically significant influence of age on attractiveness scores for both 80% lower incisors exposure images and 60% lower incisor exposure with unaltered lips (Fig. 4). Older individuals tended to assign higher scores to images with greater lower incisor exposure. Participants’ sex did not significantly influence scores.

**Table 3:** Linear regression R and R^2^ coefficients, and statistical significance (p-value ) for the entire model and for the influence of age, sex and group of evaluators (independent variables ) on the values attributed to each image. Models were run separate for each image.

	R	R^2^	F	Model p-value	Age p-value	Sex p-value	Laypersons x Dentists p-value	Laypersons x Orthodontists p-value
Reduced lower lip height								
100 Ui	0.131	0.017	0.688	0.601	0.243	0.3	0.893	0.568
80 Ui - 20 Li	0.207	0.043	1.752	0.141	0.282	0.91	0.856	0.043*
60 Ui - 40 Li	0.274	0.075	3.185	0.015*	0.115	0.623	0.388	0.006*
40 Ui - 60 Li	0.338	0.114	5.061	<0.001*	0.07	0.862	0.956	<0.001*
20 Ui - 80 Li	0.329	0.109	4.778	0.001*	0.008*	0.965	0.282	0.002*
Unaltered lower lip								
100 Ui	0.085	0.007	0.289	0.885	0.411	0.818	0.694	0.821
80 Ui - 20 Li	0.162	0.026	1.061	0.378	0.338	0.708	0.348	0.081
60 Ui - 40 Li	0.289	0.083	3.576	0.008*	0.381	0.297	0.479	0.002*
40 Ui - 60 Li	0.343	0.118	5.234	<0.001*	0.015*	0.446	0.572	0.002*
20 Ui - 80 Li	0.341	0.116	5.155	<0.001*	0.035*	0.13	0.478	0.003*

A, B, C, D - different letters indicate a statistically significant difference in the column.

## DISCUSSION

This study provides novel insights into smile attractiveness and age estimation based on varying maxillary and mandibular incisor exposure proportions, coupled with lower lip vermilion height changes. Methods included analyzing only the smile portion to avoid distractions, similar to other research.^7^ ^,^ ^8^ ^,^ ^9^ ^,^ ^11^ ^,^ ^22^ ^,^ ^26^ The online Qualtrics platform was selected for its accessibility and established performance.^27^ ^-^ ^29^

Using the VAS threshold of 5 for unattractive/attractive,^24^ maxillary incisor exposure significant impacts smile attractiveness perception. Higher exposure (100Ui and 80Ui-20Li) was consistently assessed as attractive by all evaluator groups. As mandibular incisor exposure increased, attractiveness decreased, with higher mandibular incisor exposure (40Ui-60Li and 20Ui-80Li) considered unattractive by all groups. These findings suggest universal preference for maxillary incisor visibility, possibly related to youth, health and esthetics.^16^ ^,^ ^18^

Lower lip vermilion height reduction did not significantly influence esthetic perception or age estimation. Only images with 100% upper incisor exposure showed differences between reduced and unaltered lips, possibly due more to increased gingival margin rather than lower lip dimensions, as this difference was not significant in any other proportions. Young individuals’ expected upper to lower lip vermillion height ratio ranges from 55-90%.^3^ ^,^ ^12^ ^,^ ^15^ ^,^ ^17^ ^,^ ^21^ ^,^ ^30^ ^-^ ^33^ However, literature describes decreased lower lip vermillion height as a consequence of aging, while upper lip changes are less significant.^14^ ^-^ ^17^ If increased lower lip vermilion height could distract from the unesthetic increased lower incisor exposure associated with aging, that would be valuable information for lip augmentation treatments aimed at esthetic enhancement. Nevertheless, our findings suggest that even youthful lower lip appearance with increased vermilion height could not diminish the unesthetic impact of increased lower incisor exposure in the smile. This should be carefully considered in treatment planning. Additionally, decreased lower lip vermilion height impact age perception less than lower incisor exposure extent.

Our study demonstrates that the proportion of maxillary to mandibular incisor exposure plays a significant role in age estimation. Younger individuals typically display exclusively maxillary incisors or minimal mandibular incisors during smiling.^6^ This aligns with literature suggesting that age-related changes in incisor exposure are a common phenomenon.^12^ ^,^ ^16^ ^,^ ^18^ ^-^ ^20^ However, we must consider that the amount of incisor exposure may vary depending on the situation, such as during smiling, speaking, or between static and dynamic scenarios.^16^ ^,^ ^26^ Furthermore, our results indicate that older laypersons tend to accept smiles with greater mandibular incisor exposure more readily, still perceiving them as relatively esthetic. This finding highlights the potential influence of the evaluator’s age on smile attractiveness perception and the importance of considering individual preferences in treatment planning. 

Similar to other studies, orthodontists were more critical in the smile esthetic analysis than dentists and laypersons.^5^ ^,^ ^9^ ^,^ ^34^ ^,^ ^35^ Therefore, communication between professionals and patients is extremely important in the treatment planning phase to balance expectations and ensure that the professional considers the patient’s esthetic considerations and chief complaints.

Considering clinical relevance and practical applicability of the present study, knowledge of laypersons’ perceptions may guide orthodontists’, prosthodontists’ and general practitioners’ conduct when planning vertical changes to anterior teeth.^5^ ^,^ ^16^ Careful analysis of incisors exposure during smiling, at rest, and during speech should be considered, as these changes may impact facial esthetics and age appearance.^16^ ^,^ ^26^ Additionally, professionals may use this information to enhance professional-patient communication, aligning expectations with clinically achievable outcomes.

Our findings underscore the important role of incisor display in shaping esthetic judgments and age attribution across different groups of evaluators, including orthodontists, dentists, and laypersons. Results contribute to our understanding of smile esthetics and have direct implications for clinical practice in orthodontics and esthetic dentistry, guiding treatment planning and patient communication.

Study limitations include the use of digitally manipulated static photographs, which do not fully represent the dynamic nature of smiles. Additionally, the lips, skin, and teeth features (e.g., tooth color changes, incisal wear, gingival recession, and soft-tissue aging) were characteristic of a young model, which may have influenced esthetic perception and age estimation, a multifactorial process. Therefore, the use of standardized images focusing primarily on incisor exposure may limit the generalization of age-related findings, and results should be interpreted with caution.

Although esthetic evaluation is inherently subjective, the visual analog scale (VAS) is a validated and widely accepted method in orthodontic research, demonstrating adequate reliability and sensitivity for assessing smile esthetics and perceptual differences.^24^ Future studies should include more diverse models and dynamic smile assessment to better approximate real-world conditions, as recently highlighted in the literature.^23^

## CONCLUSIONS

Greater upper incisors exposure (100% or 80%) in smiles was consistently considered more attractive by all evaluator groups. 

Greater lower incisors exposure (60% or 80%) was considered unattractive by all groups.

Orthodontists were more critical compared to dentists and laypersons.

Increased lower incisors exposure was consistently correlated with attribution of older apparent age.

Older evaluators, particularly laypersons, tended to give higher scores to smiles with greater lower incisors exposure than younger individuals.

## Data Availability

All data analyzed during this study are included in this published article. Raw data are available from the corresponding author upon reasonable request.
